# Barriers to advancing the sexual and reproductive health agenda in Latin America: a qualitative study of key informants’ perspectives

**DOI:** 10.1186/s12978-024-01927-6

**Published:** 2024-12-18

**Authors:** Juan Pedro Alonso, Cintia Cejas, Mabel Berrueta, Paula Vazquez, Gabriela Perrotta, Sandra Formia, Sofía Pirsch, Jamile Ballivian, Denise Zavala, Analía López, María Belizán

**Affiliations:** 1 National Scientific and Technical Research Council (CONICET), Buenos Aires, Argentina; 2 Instituto de Efectividad Clínica y Sanitaria (IECS), Buenos Aires, Argentina

## Abstract

**Background:**

The effective attainment of sexual, reproductive, and maternal health and rights (SRMHR) requires a holistic life-course approach. This approach should address disparities in healthcare access and rights, guarantee the delivery of high-quality care devoid of discrimination, and underscore rigorous accountability mechanisms throughout the implementation process. Latin American and Caribbean (LAC) countries face significant disparities in SRMHR within and between nations. Vulnerable populations, such as indigenous communities, Afro-descendants, LGBTQI + population, persons with disabilities, older adults, and migrants, often endure discrimination and stigmatization, severely impacting their access to healthcare and health rights. This paper presents the findings from the qualitative component of a broader mixed-methods scoping study aimed at establishing a priority research agenda to address healthcare gaps affecting the SRMHR of vulnerable populations. The qualitative component focused on identifying key challenges hindering progress in SRMHR and access to health services for these populations in the LAC region, drawing on the perspectives of key informants at both regional and national levels.

**Methods:**

Qualitative research approach employing semi-structured interviews with key informants. A purposive sample comprised of stakeholders from relevant regional organizations and local stakeholders in selected countries (Argentina, Colombia, Peru, Mexico, Guatemala, Jamaica, and Guyana), encompassing government representatives, civil organizations, and academia. A rapid content thematic analysis was conducted to analyze the data obtained from the interviews.

**Results:**

We interviewed 27 key informants in SRMHR, six at a regional level and 21 at a country level. The region faces barriers around establishing and sustaining agency agendas, such as a lack of political will, political instability, and opposition from civil society groups regarding SRMHR agendas. Policy implementation presents difficulties due to insufficient and unstable funding, weaknesses in sexual and reproductive health programs, unequal policy implementation in federal countries, and the absence of evidence-based policies. The lack of high-quality data and quality indicators poses obstacles, leading to limitations in evidence generation. Access to SRMHR services faces barriers such as the low-quality provision of services, discrepancies between legislation and effective access, insufficient healthcare resources, and resistance from certain healthcare providers.

**Conclusion:**

Addressing these multifaceted challenges will be crucial in advancing the agenda of sexual, reproductive, and maternal health rights and ensuring effective access to health services for the most vulnerable populations in the LAC region.

**Supplementary Information:**

The online version contains supplementary material available at 10.1186/s12978-024-01927-6.

## Background

Sexual, reproductive, and maternal health and rights (SRMHR) is a crucial component of universal health coverage (UHC). UHC aims to ensure all individuals and communities have access to essential health services, and achieving universal access to SRMHR is essential for progress towards UHC [1]. As countries strive for UHC, they must carefully consider the fulfillment of SRMHR requirements throughout a person's life, spanning infancy, childhood, adolescence, adulthood, and old age. These SRMHR are integral to the United Nations Sustainable Development Goals (SDGs), particularly Goal 3, which aims to ensure healthy lives and promote well-being for all at all ages, reducing maternal mortality (targets 3.1) and ensuring universal access to sexual and reproductive healthcare services (targets 3.7), and Goal 5, which focuses on achieving gender equality and empowering all women and girls, also ensuring universal access to sexual and reproductive health and reproductive rights (target 5.6) [2]. To effectively address the SRMHR of individuals, a comprehensive life course approach is essential. This approach addresses the evolving needs and challenges at different life stages, emphasizing the importance of addressing gender disparities and human rights, aiming to close equity gaps in healthcare access, and tackling systemic inequalities and power dynamics [1].

In recent decades, Latin America and the Caribbean (LAC) have made significant strides in safeguarding SRMHR by improving legal frameworks and implementing policies and programs. The Montevideo Consensus on Population and Development, adopted in 2013, has been pivotal in advancing these processes. By outlining comprehensive guidelines that emphasize human rights, gender equality, and the needs of vulnerable populations, it has provided a comprehensive regional framework for implementing and monitoring policies that promote SRMHR [3]. Despite these advancements, notable disparities persist both among and within LAC countries [3–5]. Women who face social and economic disadvantages, particularly young, impoverished, poorly educated, rural-dwelling individuals, encounter substantial challenges in accessing necessary services to prevent unintended pregnancies, maintain maternal health during pregnancy and childbirth, and ensure the well-being of their newborns. Moreover, highly vulnerable populations, including systematically marginalized groups like indigenous communities, Afro-descendants, LGBTQI + individuals, persons with disabilities, the elderly, and migrants, often experience discrimination and stigmatization, which significantly hinders their health rights and access to healthcare services [3, 6, 7]. Significant gaps also remain in ensuring sexual rights, such as barriers to access to comprehensive sexuality education (CSE), lack of legal recognition for same-sex marriages and gender identity, and widespread discrimination against LGBTIQ + populations, among others [8]. Furthermore, the strains on healthcare systems and societal responses to the COVID-19 pandemic have negatively impacted SRMH outcomes in the region and exacerbated gender-based disparities [9, 10].

Latin American and Caribbean countries can take specific actions to advance toward UHC, ensure equitable access to quality SRMH interventions, and protect health rights. These actions involve identifying and engaging stakeholders within and beyond the healthcare sector, assessing SRMHR needs, evaluating available resources and system limitations, and prioritizing interventions to be implemented across various levels of the healthcare system.

The findings presented in this paper are part of a broader mixed-methods scoping study aimed at establishing a priority research agenda to address the healthcare gaps affecting the sexual, reproductive, and maternal health and rights of vulnerable populations [11]. This qualitative component sought to uncover the key challenges in advancing the SRMHR agenda in the LAC region from the perspective of key informants at both regional and national levels.

## Methods

### Design

Between April and July 2022, we conducted semi-structured interviews with key informants at both regional and country levels. To delve into the key informants' perceptions regarding the primary gaps and barriers SRHMR, we employed a rapid qualitative appraisal research approach [12].

### Study sites and participants

The scoping study has a regional scope focusing on prioritized countries. The prioritized countries were Argentina, Colombia, Peru, Mexico, Guatemala, Jamaica, and Guyana. The choice of these countries was made in collaboration with the funding agency and aimed to encompass nations from distinct sub-regions, including South America, Central America, North America and the Caribbean.

To optimize data collection efficiency, we employed a targeted, information-oriented sampling strategy. The selection of participants was guided by their expected contributions based on their positions and expertise. The identification and choice of key informants were informed by a stakeholder mapping and analysis conducted in the earlier stages of the study, as well as input from SRMHR experts within the research team [11].

Our purposive sample encompassed organizations operating at a regional level, selected for their relevance to the study's scope and their approach to regional policies. To gather insights pertaining to the prioritized countries, our participant pool consisted of individuals from various backgrounds, including government representatives, civil organizations, and academia. We included different types of informants to capture a wide range of perspectives and ensure a nuanced understanding of the challenges to advancing SRMHR in the LAC region.

### Data collection methods

For the semi-structured interviews, we designed an interview guide that included a common set of questions for all informants, with some questions tailored for each type of informant, focused on exploring more specific challenges in research, advocacy, or policy design and implementation. This guide was designed to capture key informants’ reflections on gaps and progress in advancing the SRMHR agenda at both regional and country levels across various areas. It addressed critical topics such as legal frameworks, policy implementation, access to services, and information and research gaps. The interview guide is available in Additional file 1.

Interviews were conducted virtually using the Zoom® cloud-based video communications application. Each interview was facilitated by a tandem consisting of an experienced social scientist (MB or JPA) as the main facilitator and an expert in SMRHR (CC, GP, SF, SP, or DZ). These interviews were conducted in both Spanish and English, with durations ranging from 30 to 60 min. Audio recordings were made of each interview, and subsequently, transcribed verbatim to facilitate analysis. Prior to each interview, verbal consent was obtained from the participants.

### Data analysis

Interviews were audio recorded and transcribed verbatim. Subsequently, a rapid thematic qualitative analysis was conducted using Atlas.ti 22 software [13]. To ensure methodological rigor and comprehensiveness, a subset of the interview transcripts was independently coded by two proficient coders (MB and JPA). Employing an inductive approach, a detailed code list was iteratively developed through a systematic review of the data. This process focused on capturing the reflections and opinions of informants, from which a series of barriers to advancing the SRMHR agenda emerged. These barriers were organized into eight themes, which were subsequently grouped into three overarching categories to provide a comprehensive and structured understanding of the data. The identified barriers were initially compared at the country level and subsequently analyzed at the regional level. This approach facilitated a comprehensive exploration, allowing for the examination of variations and commonalities across diverse geographic contexts. The coding process was conducted in the original language of each interview, whether it was Spanish or English, by researchers proficient in both languages used in the study. Quotes from Spanish speakers included in the results section were translated and double-checked for accuracy.

## Results

The study included a total of 27 participants, whose characteristics are summarized in Table 1. The majority of participants were female (n = 22). We interviewed 6 key regional informants from international agencies and NGOs influential in global health and reproductive rights, particularly in LAC. At the country level, we interviewed 21 key informants. These included public sector officials involved in SRMH programs or serving as government advisors (n = 5), clinical and social science researchers in SRH, public health, women’s rights, and gender and queer studies (n = 7), and community representatives working at NGOs (n = 7), among others. The latter group advocates for women's rights, LGBTQI + communities, people with disabilities, and indigenous populations.

**Table 1:** Characteristics of participants

Characteristics	n
Gender	
Female	22
Male	5
Key informants at regional level	6
* Main role*	
Officials	5
Advocacy officer	1
* Institution*	
International agency	5
NGO	1
Key informants at country level	21
* Main role*	
Researcher	7
Program manager	5
Women/community representatives	5
Other	4
* Institution*	
University/Research institute	7
Government Ministry	5
NGO	7
Other	2
Total	27

We describe the main challenges and barriers advancing the agenda of rights and effective access to health services for the most vulnerable populations in LAC. Eight emergent themes were grouped into three main categories: political challenges; SRMHR policy design and implementation; and SRMHR services quality and access (see Table 2). We outline each theme and present illustrative verbatim quotes from key informants at regional and country level.

**Table 2:** Main categories and themes

Main categories	Themes
1. Political challenges	- Lack of political will and political instability - Obstructionist actions by civil society groups opposed to the advancement of SRMHRs
2. SRMHR policy design and implementation	- Lack of adequate and sustained funding for policy implementation - Weakness of sexual and reproductive health programs - Unequal policy implementation in federal countries - Limitations in the availability and quality of data in SRMHR
3. SRMHR services quality and access	- Low quality of SRMHR service provision - Gaps in accessing SRH services

## Political challenges

### Lack of political will and political instability

Key informants noted that governments resist legislating and implementing SRMH policies. The most opposed topics include the implementation of protocols for safe abortion and changes in the legal status of abortion (criminalized in many countries), access to emergency contraception (EC), and CSE from a gender perspective. Furthermore, participants stressed that the advance of policies centered on the LGTBIQ + population was being rejected in most countries, including the legalization of marriage or same-sex unions and the recognition of gender identity in the transgender population.

Informants from Jamaica, Guatemala, and Peru highlighted resistance to advancing the legalization of abortion—including current initiatives aimed at restricting access—as well as resistance to the implementation of EC. In Jamaica, informants noted a lack of political will to ensure access to contraceptive methods for minors. A key informant from Peru illustrated this resistance:

*We continue discussing emergency contraception in our country. All these issues have been resolved in many countries, but we are still debating in the Constitutional Court whether emergency contraception is abortive or not, after 20 years.* (Key informant at country level, Peru)

Progress in CSE with a gender and human rights perspective is unresolved in Mexico, Peru, Colombia, and Jamaica. In terms of advances in LGTBIQ + population rights, in Jamaica, sexual relations between men are criminalized, and in several countries (such as Guatemala), same-sex marriage and the recognition of gender identity are not legalized. A key informant at the regional level highlighted some of these politically sensitive topics that are prominent across various countries:

*CSE has significant political opponents. I would say that the biological component of sexual education is the one that countries really want to promote (...) The entire LGBTQ*+ *theme is a forbidden focus for many governments in the region; they do not want to talk about it.* (Key Informant at regional level)

Alternation in governance was also mentioned as a factor that hinders continuity or generates setbacks in improvements in SRMH due to the dissolution of programs or policies or to funding discontinuity. In Peru, the political instability and frequent government transitions were cited as impediments to the sustained continuity of healthcare policies, including those related to SRMHR. Informants from Guatemala highlighted the absence of a national health law as a key barrier to ensuring continuity in SRMH policies:

*The problems we face are related to macro laws, for example, we do not have a National Health Law. So, every four years, with a new government, the approach to health work changes as the government pleases, even though the problems remain the same. What the previous government did is not useful.* (Key informant at national level, Guatemala)

### Obstructionist actions by civil society groups opposed to the advancement of SRMHRs

One crucial barrier mentioned by informants was the obstructionist actions conducted by civil society groups opposing the progress of the SRMHR agenda and undermining individual rights. The groups' actions include lobbying activities at the political level to influence legislation, judicialization strategies, and pressure to block the implementation of policies and programs, mainly in CSE and abortion. An informant from Mexico illustrates the actions of these groups opposing advances in SRHR initiatives:

*In Mexico, there is a conservative movement or a series of well-funded and well-organized conservative groups that react immediately to any progress related to sexual and reproductive rights, particularly abortion.* (Key informant at a country level, Mexico)

Informants from Guatemala mentioned legislative initiatives to reverse progress made on SRMHR promoted by these groups. In Peru, the judicialization of the abortion protocol and the resistance to EC were mentioned. Resistance to CSE implementation represents a prominent point of contention within these groups. Informants from Guatemala, Colombia, and Mexico underscored the challenge of integrating SRMHR priorities within the education ministries, thus impeding advancements in sexual education implementation. To illustrate this common challenge, an informant from Colombia described barriers to implementing CSE:

*CSE is one of the challenges in this country because every time an attempt has been made to implement a comprehensive sexual education policy, it has been very difficult within the Ministry of Education. I believe it is one of the most resistant institutions. (Key informant at a country level, Colombia)*

## SRMHR policy design and implementation

### Lack of adequate and sustained funding for policy implementation

Another challenge mentioned by key informants in some countries was the inadequate and unsustainable funding to ensure the implementation of SRMHR policies. Lack of supplies to ensure SRMHR, such as limitations in the availability of some types of contraceptives and medical abortion supplies, as well as the lack of funding for human resources were mentioned. Due to the COVID-19 pandemic, the budget for SRMH has been considerably reduced in several countries in the region, as it was not considered a priority in the response strategies to the health emergency. A key informant from Guatemala highlighted a common barrier by pointing out financing challenges and a lack of appropriate supplies:

*Another problem has to do with financing. We do not exceed spending 1.2 or 1.3 percent of the GDP budget on health. Therefore, health services are consistently under-supplied, especially those at the primary and secondary levels. (Key informant at a country level, Guatemala)*

At a regional level, some key informants pointed out the reduction of government and international donor funding for SRMHR in the LAC region as a result of a reallocation of resources towards priority countries or areas, such as the migration crises in the region or the global health emergency caused by the COVID-19 pandemic. As a key informant at the regional level stated: *"We no longer have donors. We are a region that has graduated from international donors. The region is no longer prioritized."* This reduction was also due to government decisions in donor countries, such as the US and the UK, to withdraw funding from international UN agencies. They also highlighted donors’ capacity to influence the agendas and activities of regional and international organizations, for example, by limiting actions related to the advancement of abortion laws or initiatives. This is often done by restricting funding for organizations or programs that promote abortion services.

### Weakness of sexual and reproductive health programs

Informants from some countries, such as Guatemala, Peru and Guyana, highlighted the weakness of SRMH programs as a barrier to guaranteeing access to SRMHR. The relegated position of the programs in the organizational structure of the Ministries of Health (MoH) in some countries, the scarce funding for the design and implementation of policies, and the lack of human resources allocated to the area at the managerial level were emphasized. As noted by an informant from Guyana: *"We have very good laws on paper in Guyana, but certainly, the implementation of policies and ensuring the resources to carry them out is insufficient."* An informant from Peru also illustrated some of the challenges identified, such as the lack of political influence of SRMH programs and the absence of coordination with HIV/AIDS programs:

*At the organizational level, the reproductive health department is very low in the hierarchy of the Ministry of Health; it does not have political, institutional, or budgetary weight. In Peru, the SRH department is separate from the HIV department. (Key informant at national level, Peru)*

Another aspect highlighted as a barrier in some countries was the prioritization of maternal health in the policies and budget of SRH programs. Another barrier was the low governance capacity of the programs to monitor and guarantee the implementation of policies and regulations by the different health sub-systems and providers. Informants from Colombia, for example, referred to the limited capacity of the national program to monitor health insurers to ensure effective access to contraception and abortion:

*There are no consequences when barriers are imposed by providers, or they’re not strong enough. Who is in charge of monitoring the implementation of sexual and reproductive health policies? The Ministry of Health. That monitoring is a challenge. (Key informant at country level, Colombia)*

Several factors were identified as contributing to the fragility and limited prioritization of SRMH programs at the national level. These include a lack of political will and limited understanding among authorities of the adverse impacts of neglecting SRMH on development, poverty, and inequality, among others.

### Unequal policy implementation across states in national federations

A challenge highlighted by informants from countries with federal government systems, such as Argentina and Mexico, was the unequal implementation of policies in the different states or provinces. In Mexico, informants highlighted the challenge of legalizing access to legal abortion at the federal level and reducing the gaps in access to this right at the sub-national level. In Argentina, informants mentioned inequality in the implementation of different policies, such as access to legal abortion, CSE, and access to contraceptives, due to resistance in some provinces. As an informant from Argentina explained:

*The country faces the major challenge that a national law doesn't necessarily ensure its implementation as public policy in the provinces. This is because, in terms of management and administration, the provinces could choose not to adhere to the law or limits its implementation. (Key informant at a national level, Argentina)*

Also related to government systems, informants from Colombia highlighted the role of the Supreme Court of Justice, whose jurisprudence carries the legal weight of legislation, in advancing the SRMHR agenda, as well as the challenges posed for implementation. As one informant explained, referring to the recent decriminalization of abortion up to 24 weeks by the Colombian Supreme Court: *"One of the gaps and challenges we face is turning those judicial decisions, particularly regarding abortion, into concrete actions that help implement those rulings".*

### Limitations in the availability and quality of data in SRMHR

The low quality of data, the difficulty in accessing, and the limited use of data for policy design and evaluation were highlighted by key informants at a regional and national level. Among the barriers to obtaining quality and accessible data timely are the weakness of the information systems, deficiencies in the records at a provider level, fragmented health systems, the lack of political will, and the lack of an institutional culture for data-based decision-making by health authorities.

Informants from Argentina and Mexico stressed the need for more information from private health and social security subsystems. Similarly, informants from Colombia, Peru, and Guatemala mentioned quality problems, such as a need for more consistency between data reported at the national and sub-national levels due to the fragmentation of registration systems. In Peru, the coexistence of many information systems under different administrators (epidemiology directorate, registries under specific programs, etc.) was mentioned, leading to duplication of resources and fragmentation of registries. An informant from Peru illustrated some of these challenges:

*The information systems in the country are like the health system: completely fragmented. Access to data is not timely, and the data are not used to make decisions. (Key informant at a local level, Peru)*

Limited data and delays in the public dissemination of data were also mentioned. In Guatemala and Jamaica, for example, accessing data produced by the MoH poses a considerable challenge. An informant from a Guatemala-based NGO noted that, at times, they are forced to *“access data through the Public Information Access Law”.*

Another central area for improvement in data production is the need for disaggregated data by equity strata, limiting the evidence generation from an intersectionality perspective and the design of targeted policies. Information systems usually do not include variables for measuring the differential impact in vulnerable populations. The lack of evidence regarding access to SRMH services for vulnerable populations, such as rural, indigenous, and Afro-descendant populations, was emphasized by participants. *As noted by a regional key informant:*

*We don't have the data we need disaggregated, for example, by equity stratifiers. We don't have them; we don't have data* broken by rural, urban*... The data generated by administrative systems lack that complete information, so there is a significant gap in terms of data and evidence generation. (Key informant at a regional level)*

Additionally, the region faces a deficiency in its capacity to generate evidence related to SRMHR, and the existing body of evidence is disproportionately concentrated in a select few countries. This is interpreted as the result of insufficient resources allocated to promote research and the need to strengthen research capacities at the country level. The available evidence used for policy design usually comes from central and high-income countries, whose research is not necessarily adapted to local needs:

*I would say that evidence is being produced, but generally the problem is that evidence is not produced equally across all the countries in the region. So, a lot of evidence used is produced primarily in high-income countries. (Key informant at regional level)*

## SRMHR services quality and access

### Low quality of SRMHR service provision

The lack of trained human resources significantly affects the quality of services. Key informants from most countries noted deficiencies in the training of health personnel and stressed the lack of training in gender and human rights approaches. Peru and Mexico also emphasized the limited level of training of health providers in intercultural health for the care of indigenous or rural populations. Informants from several countries mentioned the continuity of paternalistic models of healthcare delivery. Informants from Colombia and Argentina highlighted the challenge of including or strengthening undergraduate and postgraduate training of professionals in SRMHR issues, particularly in abortion, human rights, and gender. This fragment from an informant in Colombia illustrates the deficiencies in training:

*There are women with unwanted pregnancies due to poor counseling or negligence in contraceptive care. The biggest barrier is not in a normative or legislative vacuum; it lies in a gap in the training of professionals in human rights and a gender perspective that prevents all these norms from reaching their beneficiaries. (Key informant at a national level, Colombia)*

Some informants pointed out the need to improve the provision of SRMH services by implementing or reinforcing targeted policies for specific populations. Informants from Peru, Guatemala, and Mexico pointed out the lack of differentiated policies and programs for the care of indigenous, rural, and Afro-descendant people with an intercultural approach. The need for cultural adaptations of SRMH services to guarantee effective access for these populations was highlighted. As noted by a Mexican informant:

*There is a fundamental gap in the provision of services for rural women, and even more so for Indigenous women, particularly in access to modern family planning methods. I believe this highlights an issue that needs to be addressed across all areas related to SRHRs: the need to understand and consider local cultures. (Key informant at a national level, Mexico)*

Resistance from healthcare providers to implement SRMH practices was also mentioned as a challenge to the quality-of-service provision. Participants mentioned barriers to safe abortion care in health facilities due to predominant restrictive interpretations of the legislation among health personnel. According to informants, these practices limit effective access to SRHR as established in the legislation. Some professionals refuse to perform recommended practices, such as intrauterine device (IUD) insertion procedures, as illustrated by an informant from a regional organization:

*The IU (intrauterine device) is a very inexpensive, excellent method, and healthcare providers discard it. First, because they don't know how to insert it, as there is no longer training in universities. The loss of competencies in the training in some of these areas is significant in the region. (Key informant at regional level)*

### Gaps in accessing SRH services

Key informants highlighted significant disparities in access to SRMH services, particularly affecting vulnerable populations such as adolescents, women in rural areas, indigenous communities, migrants, and LGBTIQ + individuals. These groups face greater challenges in accessing a broad spectrum of services, including contraceptives, prenatal care, childbirth care, timely diagnosis of sexually transmitted infections, and abortion, among others.

Among the main barriers to access SRMH services, language barriers, geographic barriers to access to healthcare providers, cultural barriers, community resistance, displacement of nomadic groups, and discrimination by health providers were noted. As an example of cultural barriers, informants from Guatemala, Peru, and Colombia identified deficiencies in implementing culturally relevant childbirth care and situations of mistreatment and obstetric violence during childbirth. In rural areas, there are noticeable geographic barriers to access to timely referral systems for at-risk pregnancies, delays or reluctance of rural and indigenous women to go to health centers, and a lack of community support for women. As noted by an informant from Peru:

*Not only are rural women farther away, making access to services difficult, but we also need to have cultural adaptations in services so that they can truly receive the information and services they need, and that is practically forgotten. (Key informant at a national level, Peru)*

The migrant and the LGBTIQ + population have less access to SRMHR services and experience discrimination in health services, stigmatization, and criminalization. Concerning this, key informants from most countries noted deficiencies in the training of health personnel and stressed the lack of training in gender and human rights approaches. An informant from Jamaica explained the barriers faced by LGTBQI + community:

*The LGBTQ community has limited access to health care. They can't access services due to discrimination. Lesbian, bisexual, and queer women also face a lot of challenges because there's another conversation around their sexual health needs and their reproductive health needs. (Key informant at a national level, Jamaica)*

Key informants from all countries also identified challenges related to accessing services tailored to adolescents, such as counseling, access to long-acting contraceptives, and abortion, as well as limited access to CSE. As noted by an informant from a regional organization:

*There are countries that recognize people's right to freely access and decide on a contraceptive method, but when you examine how this is implemented, barriers related to age become evident. For example, in several countries, healthcare providers decide not to offer an intrauterine device to an adolescent girl, regardless of WHO recommendations, or refuse to provide hormonal contraceptive methods. (Key informant at regional level)*

## Discussion

Through semi-structured interviews with regional and country-level key informants, we identified key political challenges hindering progress in the SRMHR agenda, barriers to policy implementation, and obstacles to ensuring effective access and quality service provision for vulnerable populations in LAC.

As in many other regions, political barriers remain a significant obstacle to the progress and sustainability of SRMHR in the region. While there has been progress in some LAC countries toward compliance with the Montevideo Consensus and international recommendations [3, 14], our findings highlight that the national contexts exhibit varying degrees of political will and adherence to these commitments. Over the past decade, legislative advances in certain countries have included the recognition of same-sex marriage and adoption, the acknowledgment of gender identity on national ID cards, and the enactment of anti-discrimination laws [8, 15, 16]. However, the legal frameworks and policies governing SRMHR remain heterogeneous and, in many cases, require updating [5, 17].

In several countries, governments have been reluctant to advance legislation and implement policies addressing socially divisive issues such as the legal status of abortion, CSE, and rights-based policies for the LGTBIQ + population. These challenges are not unique to LAC; similar political resistance to advancing the rights of sexual and gender minorities has been documented in other regions [18, 19].

Moreover, the alternation of governments poses further challenges to the continuity of SRMHR initiatives, often leading to setbacks. For instance, in Guatemala, the arrival of conservative political parties has restricted access to SRMHR services [17, 20]. Similarly, in Peru, a climate of political instability and frequent government changes has hindered the continuity of health policies, including those related to SRMHR [21]. These findings underscore the complex interplay between political dynamics and the effective implementation of the SRMHR agenda in the region.

Resistance from certain organized civil society groups has posed challenges to the progression of SRMHR in the region. These groups often engage in political lobbying or appeal to the courts, as documented in various LAC countries [22]. Their efforts are particularly directed against the legalization or decriminalization of abortion [23] and the implementation of CSE with a gender perspective [24, 25]. This resistance reflects ongoing challenges in advancing SRMHR policies amidst competing social and political pressures.

There are challenges related to policy implementation within many countries. Insufficient and unstable funding for SRMH policy execution is a common barrier in the region; a common challenge for the SRH programs in low and middle-income countries [5, 18]. This lack of resources creates significant obstacles to the effective functioning of programs and access to SRMHR services at the health service level. For example, the unavailability of free contraceptives from many providers compels individuals to pay out of pocket, effectively excluding the most vulnerable groups. Furthermore, in some countries with CSE programs, insufficient planning for the allocation of human and material resources has been documented [26, 27].

The findings of this study also highlight that reductions in government and international donor funding for the LAC region have impacted policy implementation efforts, with middle-income countries being particularly affected due to their lower prioritization. Moreover, SRMH was overshadowed by other health priorities, such as the migration crises in the region or the global health emergency caused by the COVID-19 pandemic. The strains on healthcare systems and the societal responses to the COVID-19 pandemic had a negative impact on the regional SRMH indicators and exacerbated gender-based disparities [10, 28].

Adequate and sustained funding by international donors is a significant source of financing for SRMH services and interventions in LMICs, as other review studies show [18, 29]. Improperly managed donor transitions in middle-income countries can pose significant challenges to global health progress; however, there is a need for a more comprehensive evaluation of how these transitions can affect health systems in those countries [30].

A key finding of this study is the gap in the development of policies based on local evidence, driven by the absence of high-quality data in the region, as well as challenges related to research capacity and funding. Weaknesses in record keeping, problems with the information systems used, and low priority given to the production of primary data affect quality data generation in a regular and timely manner. Information gaps include data related to prenatal care (for example, vertical transmission of congenital syphilis); abortion (for instance, clandestine and unsafe abortions in countries that penalize the practice, unmet demand, access to drugs, and repeat abortions); and gender-based violence (for example, evidence about the effectiveness of actions to reduce it) [31–33].

Several gaps in access to SRMH services have been identified, with these disparities being particularly pronounced among vulnerable populations, such as indigenous and rural women, LGBTQI + individuals, and adolescents. A significant barrier for adolescents was highlighted, including limited access to contraceptive methods (e.g., IDU) and challenges in accessing CSE. These barriers leave young people less equipped to make informed decisions about their sexual and reproductive health, potentially leading to long-term negative impacts on their health and well-being [1, 34, 35].

In the context of the health emergency caused by the COVID-19 pandemic, access barriers increased due to supply shortages. The closure or limitation of services (such as prenatal check-ups, family planning, and provision of contraceptive methods, among others), the low prioritization of SRHMR in health emergency response strategies, and the reduction in budgets allocated to the area had a negative impact on SRMHR [36, 37]. Normal levels of service provision have not been reestablished in some countries by the time of the study.

The lack of trained human resources was also identified as a key factor contributing to both access gaps and the low quality of SRMHR service provision. The need to improve SRMH services to meet gender and human rights standards for quality of care has been identified as a common challenge in LMICs [38].

Since SRMHR services are essential to UHC, achieving UHC seems an elusive milestone until access to quality SRMHR services in Latin America are guaranteed. States bear the primary responsibility to address systemic barriers by integrating SRMHR into national health systems through evidence-informed policies, equitable resource allocation, and targeted efforts to reduce inequities. Research underscores the need to improve access among vulnerable populations, ensuring that services reach those most affected by social, economic, and geographic disparities. These measures are indispensable for advancing both health equity and the broader goal of UHC.

The study has significant limitations. As a qualitative rapid assessment, the study was based on a limited number of informants at the regional and country level. Although efforts were made in each country to include different informant profiles to capture other points of view, including decision-makers, non-governmental organizations, and researchers with expertise in the SRMHR field, this was only possible in some countries, and some stakeholders were under-represented in the sample. Incorporating representatives of civil society and vulnerable groups could have contributed to identifying barriers and challenges from the perspective of those targeted by SRMHR policies. Despite these limitations, the study provides relevant qualitative evidence on the main barriers and challenges to advancing the SRMHR agenda in the region.

## Conclusion

Effectively tackling the intricate challenges highlighted in this study will play a pivotal role in progressing the objectives of sexual, reproductive, and maternal health rights and in guaranteeing equitable health service access for the most vulnerable populations in the LAC region. Based on the identified challenges, several key policy actions emerge. Strengthening political commitment and ensuring stability through binding legislative frameworks is crucial to advance and sustain SRMHR in LAC. Additionally, increasing and stabilizing funding for SRMHR programs is essential for consistent support. Enhancing data collection and utilization by developing robust health information systems will inform policy decisions, monitor progress, and identify gaps, allowing for targeted interventions. Improving service quality and accessibility through training healthcare providers in gender, cultural competency, and human rights will ensure inclusive and culturally appropriate care. Ultimately, the role of the state in guaranteeing these actions is fundamental, as it must ensure that SRMHR services are integrated into the broader health system and that policies prioritize the needs of marginalized communities to achieve UHC.

## Supplementary Information

Additional file 1.

## Data Availability

Data form the interviews cannot be publicly shared to protect participants' confidentiality, as sharing them could potentially lead to deductive disclosure of their identities. Key informants who participated in the study are well-known researchers or decision-makers in their countries or fields of expertise, and the details provided in the interview could violate the anonymity and confidentiality of their participation. However, excerpts of the interviews are available from the authors upon reasonable request if they cannot be shared publicly.

## Abbreviations

CSE: Comprehensive sexuality education
EC: Emergency contraception
LAC: Latin American and Caribbean
LGBTQI +: People who have identified themselves as lesbian, gay, bisexual, transgender, intersex or questioning and others
MoH: Ministry of Health
NGOs: Non-governmental organization
SDGs: United Nations Sustainable Development Goals
SRMH: Sexual, reproductive and maternal health
SRMHR: Sexual, reproductive, and maternal health and rights
UHC: Universal health coverage
UN: United Nations
