# Supplementary material for: Barriers to advancing the sexual and reproductive health agenda in Latin America: a qualitative study of key informants’ perspectives
Source: Reprod Health. 2024 Dec 18;21:187. doi: 10.1186/s12978-024-01927-6 (PMC11657184; doi:10.1186/s12978-024-01927-6)
Supplement: Supplementary file 1 — Additional file 1. [file 12978_2024_1927_MOESM1_ESM.pdf]

Additional File 1 - Interview guide with key informants at country and regional levels

1. I would like to ask you for a brief introduction: could you share your education/background, current position, and the tasks you perform?
2. From your understanding, what are the main challenges in your country / the LAC region regarding legislation and regulation on SRMHR?
3. What are the primary challenges in implementing policies that enable effective access to SRMH services in your country / the LAC region?
4. In relation to more vulnerable groups (such as indigenous and Afro-descendant populations, LGBTQI+ individuals, migrants, people with disabilities, girls, and adolescents), what are the challenges in guaranteeing the SRHR of these populations?
5. Do you believe that the necessary information on SRMH is being produced, or are there significant gaps in SRH information in your country / the region?
6. What do you consider the main research gaps in SRMH?
7. What are the main barriers to conducting research on these issues?
8. What lessons can be drawn from the processes of advancing SRMHR in your country / the LAC region?
9. Is there anything else you would like to add regarding the issues we discussed?
